# Molecular Pathways Associated with Kallikrein 6 Overexpression in Colorectal Cancer

**DOI:** 10.3390/genes12050749

**Published:** 2021-05-16

**Authors:** Ritu Pandey, Muhan Zhou, Yuliang Chen, Dalila Darmoul, Conner C. Kisiel, Valentine N. Nfonsam, Natalia A. Ignatenko

**Affiliations:** 1 Department of Cellular and Molecular Medicine, University of Arizona, Tucson, AZ 85721, USA; nai@email.arizona.edu; 2 University of Arizona Cancer Center, University of Arizona, Tucson, AZ 85724, USA; cckisiel@email.arizona.edu; 3 Bioinformatics Shared Resource, University of Arizona Cancer Center, Tucson, AZ 85724, USA; mhz@email.arizona.edu (M.Z.); yuliangchen@email.arizona.edu (Y.C.); 4 Institut National de la Santé et de la Recherche Médicale (INSERM), Université de Paris, Lariboisière Hospital, 75010 Paris, France; dalila.darmoul@inserm.fr; 5 Department of Surgery, Section of Surgical Oncology, University of Arizona, Tucson, AZ 85724, USA; vnfonsam@surgery.arizona.edu

**Keywords:** colorectal cancer, kallikrein 6, TCGA, gene set enrichment analysis, K-RAS-oncogene, regulatory pathways, organoid culture

## Abstract

Colorectal cancer (CRC) remains one of the leading causes of cancer-related death worldwide. The high mortality of CRC is related to its ability to metastasize to distant organs. The kallikrein-related peptidase Kallikrein 6 (KLK6) is overexpressed in CRC and contributes to cancer cell invasion and metastasis. The goal of this study was to identify KLK6-associated markers for the CRC prognosis and treatment. Tumor Samples from the CRC patients with significantly elevated *KLK6* transcript levels were identified in the RNA-Seq data from Cancer Genome Atlas (TCGA) and their expression profiles were evaluated using Gene Ontology (GO), Phenotype and Reactome enrichment, and protein interaction methods. KLK6-high cases had a distinct spectrum of mutations in titin (*TTN*), *APC*, *K-RAS*, and *MUC16* genes. Differentially expressed genes (DEGs) found in the KLK6-overexpressing CRCs were associated with cell signaling, extracellular matrix organization, and cell communication regulatory pathways. The top KLK6-interaction partners were found to be the members of kallikrein family (KLK7, KLK8, KLK10), extracellular matrix associated proteins (keratins, integrins, small proline rich repeat, S100A families) and TGF-β, FOS, and Ser/Thr protein kinase signaling pathways. Expression of selected KLK6-associated genes was validated in a subset of paired normal and tumor CRC patient-derived organoid cultures. The performed analyses identified KLK6 itself and a set of genes, which are co-expressed with KLK6, as potential clinical biomarkers for the management of the CRC disease.

## 1. Introduction

One of the key hallmarks of cancer is an ability of transformed cells to invade surrounding tissues, which occurs through induction of cell- and tissue-specific signaling pathways [1]. Proteases, which catalyze the hydrolysis of peptide bonds, have long been associated with colon cancer progression because of their ability to degrade extracellular matrices. Tissue kallikrein-related peptidases (KLK) are a subgroup of serine proteases, known to serve a variety of functions, ranging from the normal physiological processes to different disease conditions, including cancer [2]. Several members of kallikrein family are overexpressed in colon cancer patients, i.e., KLK4, KLK6, KLK7, KLK10, and KLK14 [3,4,5,6]. Because of association of kallikreins with malignancy they have been proposed as potential diagnostic/prognostic and therapeutic markers [7]. Particularly, *KLK6* mRNA levels correlated with serosal invasion, liver metastasis, advanced Duke’s stage, and overall poor patient prognosis [8]. Presence of *KLK6* transcripts in lymph nodes of colorectal cancer (CRC) patients was associated with the shorter average survival time after surgery and was more sensitive indicator of poor prognosis than carcinoembryonic antigen (CEA) [9]. As we previously reported, KLK6 expression in colon cancer can be induced by the major colon cancer driver gene, oncogenic *K-RAS*, while the knockdown of *KLK6* in colon cancer cells leads to suppression of their invasive and metastatic properties [10,11,12].

The aim of our study was to analyze The Cancer Genome Atlas (TCGA) RNA-Seq data from the colorectal cancer samples with high *KLK6* expression with the goal to define KLK6-specific gene signature in the CRC for identification of potential markers of tumor progression in colorectal metastatic disease. Our analysis revealed a set of genes, which were differentially expressed in the CRC tumors, which overexpressed *KLK6*. The set includes genes involved in the regulation of cell signaling, cell-cell communication and proteolysis. The results were confirmed in another GEO dataset (GSE39582). We also analyzed the expression of *KLK6* and selected co-expressed genes in the subset of colonic organoids developed from the patients’ surgical tissue (paired normal and tumor cultures).

## 2. Materials and Methods

### 2.1. RNA-Seq Data Processing

Colorectal Cancer samples from The Cancer Genome atlas (TCGA) were downloaded from Genomic Data Common Website (GDC at https://gdc.cancer.gov/, accessed on 2019). A total of 480 tumor samples were assayed for transcription. We used the GDC data transfer tool client and GDC API to download all the RNA-seq raw counts data, metadata, and available clinical data. Data was analyzed using R (v 3.6.3) and open source methods implemented in R. Raw HTSEQ counts data was normalized using Variance Stabilizing Transformation (VST) method [13] and data was processed further. Z scores were calculated for *KLK6* expression across samples and high (*N* = 16) and low (*N* = 7) groups were defined by ≥1.96 and <−1.96 cutoff values, they represent statistically significant outliers. Differential analysis was done using the DESEQ2 [14]. Pearson correlation coefficient was used to identify genes that correlate with *KLK6* amongst high and low samples. GEO dataset GSE39582 was downloaded and the array data for 566 colon tumor samples was analyzed for high *KLK6* expression. 30 samples were found to be outliers with high *KLK6* expression (z score >1.96). Genes results from TCGA high KLK6 tumors were compared to high KLK6 tumors in GSE39582.

### 2.2. Survival Analysis of Samples with Overexpressed KLK6

The normalized data was combined with clinical data for high KLK6 samples (16), low KLK6 (7) vs. other groups (*N* = 457) for TCGA data. The overall survival (OS) in KLK6 high samples compared to other samples was done by utilizing Kaplan-Meier survival plot. GEO dataset was divided into top and bottom quartiles based on KLK6 expression and for high KLK6 samples (30) vs. others (536). Logrank test was applied to assess the statistical significance between the survival curves. The survdiff function under rms library in R (version 3.6.3) was used to perform the log-rank test.

### 2.3. Enrichment Analysis

Differentially expressed genes (DEG) were analyzed for Gene Ontology using enrichGO from Cluster Profiler and Reactome pathways gene enrichment analysis using enrichPathway from ReactomePA in Bioconductor. Protein interaction data was analyzed using String data resource [15]. The list of differential protein coding genes between high and low KLK6 were searched against Stringdb. The network was filtered for first interacting partners of kallikreins and their associations. Plots: All plots and heatmaps were generated using ggplot2 library and other data visualization packages in R.

### 2.4. CRC Patient-Derived Organoid Cultures

The 3D cultures were generated from the fresh surgical material collected as part of the University of Arizona Cancer Center (UACC) Tissue Acquisition and Molecular Analysis Shared Resource (TACMASR) Biorepository efforts. The CRC patients were consented for the tissue collection and all individual patient-related reports and data were de-identified before processing for the tissue biobanking and developing of organoid cultures. Because all information related to the used surgical material was de-identified and did not include any protected health information (PHI), it is not considered a clinical research according on the Human Subjects Protection Program (HSPP) determination. Tissue collection was approved by the Biorepository Oversight Committee at the UACC. The paired normal and tumor specimens, retrieved from the resected surgical material, were verified by a certified pathologist. The collected material was placed in the cold Advanced DMEM media and transported to the lab on ice, where they were processed for establishment of organoid cultures as described elsewhere [16]. The organoid cultures generated from paired normal and tumor tissue samples of eight CRC patients were analyzed in this study. 

### 2.5. Quantitative Reverse-Transcription Polymerase Chain Reaction 

Organoid cultures were washed with AdDMEM/F12 media (Thermo Fisher Scientific. Onc. Carlsbad, CA, USA) once and processed for total RNA isolation using RNeasy^®^ Mini Kit (Qiagen GmbH, Hilden, Germany, Cat. 74104) according to the manufacturer instructions. Reverse transcription to produce cDNA template was completed using the Applied Biosystems High Capacity cDNA Reverse Transcription Kit (Part #4368814). Quantitative Reverse-Transcription Polymerase Chain reaction (qRT-PCR) was performed using TaqMan® probes (Applied Biosystems, Thermo Fisher Scientific, Inc., Waltham, MA, USA) specific for the mRNAs of interest: KLK6 (Hs00160519_ml), keratin 6A (KRT6A) (Hs00749101_s1), keratin 6B (KRT6B) (Hs04194231_s1), FOSL1 (Hs04187685_m1), MYC (Hs00153408_m1), HMGA2 (Hs04397751_ml). 0.2 µg of total RNA was reversed transcribed into cDNA in a 20 µL reaction with random hexamers under thermal condition recommended by the protocol. Real-time PCR amplification was performed with the ABI PRISM 7700 SDS instrument (Applied Biosystems, Life Technologies, Inc., Carlsbad, CA, USA), under the universal thermal cycling conditions recommended by the Assay-on-Demand products protocol. Negative controls without template were included in each plate to monitor potential PCR contamination. The expression of genes was tested in triplicate and each reaction was run in duplicate. To determine the relative expression level of each target gene, the comparative *CT* method was used. The *CT* value of the target gene was normalized by the endogenous reference β2-microglobin (β2M, FAM (Hs99999907_m1)). The relative expression of each target gene was calculated via the equation 2^−Δ*C*^*_T_* where Δ*C_T_* = *C_T_*_(target)_ − *C_T_*_(endogenous control)_.

### 2.6. Enzyme-Linked Immunosorbent Assay (ELISA) for KLK6

Organoid cultures were seeded at ~1000 cells per 25 µL of growth factor reduced, phenol free Matrigel (Corning^®^Matrigel^®^Matrix Cat.# CB40230C) per well in 48-well plates in 250 µL/well organoid culture medium. Conditioned media was collected from the cultured organoids on days 2, 4, 7, and 10 after subculture. KLK6 levels were expressed as picograms per milliliter of media. An ELISA kit for the detection of human KLK6 in conditioned media was obtained from Boster Biological Technology Co, Ltd. (Pleasanton, CA, USA, Cat# EK0818). The detection range for this kit is 0.078–5 ng/mL. KLK6 antigen levels are expressed as nanograms per milliliter. The assay was performed according to manufacturer’s instructions. The plate was read at 490 nm within 30 min of assay ending on a Synergy 2 Multi-Detection Microplate Reader (Bio-Tek Instruments, Inc., Winooski, VT, USA). In each experiment samples were analyzed in triplicates and all experiments were repeated two or three times. 

## 3. Results

### 3.1. Pattern of Kallikrein Related Peptidases Family Expression in CRC Patients

**Figure 1:**
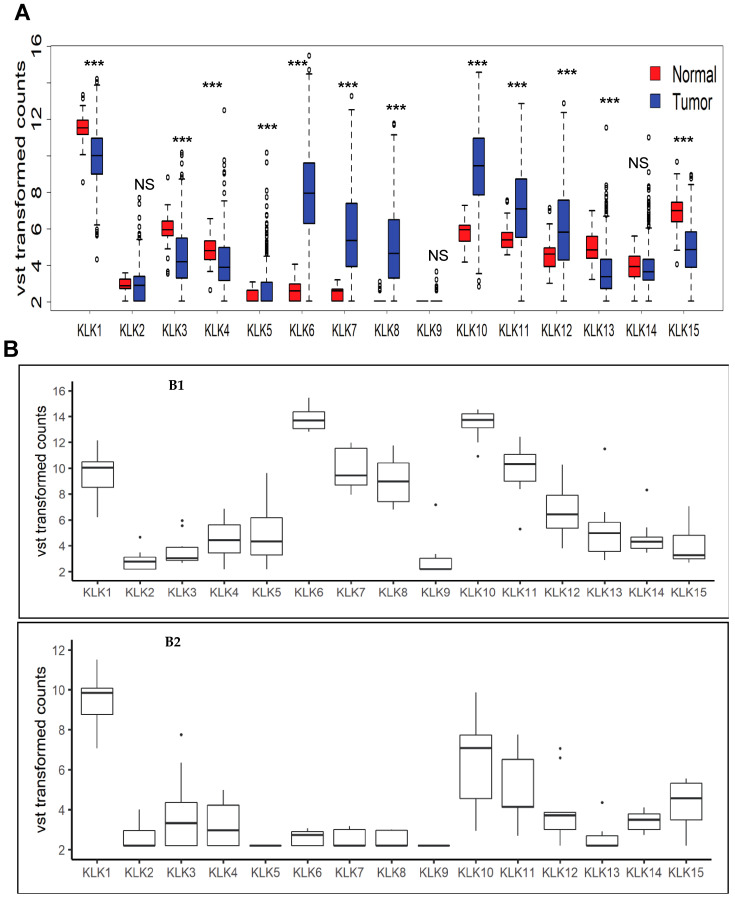
Expression of all members of Kallikrein related peptidase family in colon adenocarcinomas TCGA patient samples. (**A**) Box plot shows expression of KLK gene family normalized by Variance Stabilizing Transformation (VST) method in colon tumors and their relative expression in normal tissues. *** = *p* values < 0.0001, NS = Not Significant. (**B**) Box plots of KLK family from the CRC patient samples that have been identified as KLK6-high group and KLK6-low group. B1: Box plot of expression of all Kallikrein related peptidases in samples with high KLK6 expression (KLK6-high group) and B2: Box plot of expression of all Kallikrein related peptidases in samples with low KLK6 expression (KLK6-low group).

In this study we initially analyzed the relative expression pattern of the members of kallikrein-related peptidase genes family in 480 CRC tumor samples from TCGA. We noted that the transcript levels of a majority of kallikrein-related peptidases vary significantly in the normal and tumor samples amongst the CRC patients (Figure 1A). The normalized transcript levels of *KLK5, 6, 7, 8, 10, 11*, and *12* genes were elevated in tumor samples, compared to normal ones while levels of *KLK1, 3, 4, 13*, and *15* were found to be lower in tumors, compared to normal tissues. The levels of *KLK2, 9,* and *14* gene transcripts were not altered in tumor samples. Focusing on KLK6, we further selected the samples that have unusually high or low *KLK6* expression. We normalized row data using Variance Stabilizing Transformation (VST) method [13] and identified outlier samples with >±1.96 z score as samples with high and low *KLK6* expression, respectively. Using these criteria, sixteen samples were identified as high KLK6 expressers, and seven samples were selected as low KLK6 expressers. These samples were outliers with respect to KLK6 expression and the intent was to compare two distinct expression group of samples for identification of KLK6 expression associated biomarkers in tumor samples. These groups were named as KLK6-high and KLK6-low groups, respectively. The initial analysis revealed the distinct pattern of expression of KLK family members in KLK6-high group. Relative expression of kallikreins family members was plotted for these two groups (Figure 1B1-KLK6 -high group, and Figure 1B2-KLK6-low group). In KLK6-high group, we observed a particularly high *KLK10* co-expression, followed by *KLK7* and *KLK8*. We also noted that *KLK10* transcript levels were significantly higher than *KLK6, KLK7*, and *KLK8* in normal tissues (Figure 1A), and *KLK10* was elevated in tumor samples in KLK6-low group but to a lesser degree than in KLK6-high samples (Figure 1B2).

### 3.2. Clinical and Molecular Characterization of KLK6-High Expressing Samples

The tumors in TCGA patients with KLK6-high and KLK6-low groups were identified as colon adenocarcinomas. In KLK6-high group, the number of patients with the advanced stages of the disease constituted more than third of cases (stage III and stage IV: 18.8% and 25%, respectively). In contrast, more than half of the patients in KLK6-low group had the early stages of the disease (stage I and stage II: 28.8% and 43%, respectively), with no stage IV patients (Table 1). The CRC tumors with high and low KLK6 expression were stratified into the microsatellite stable (MSS) and microsatellite instable (MSI) molecular subtypes as defined in other TCGA studies [17]. Both groups had comparable numbers of MSS tumors. The MSI tumors with high (MSI-H) or low (MSI-L) number of mutations were represented equally in the KLK6 high and KLK6 low groups (Table 1). Similarly, no association or correlation with any particular CMS (Consensus Molecular Subtype) [18] (Figure S1) was found for high KLK6 expression in tumors.

We further determined the spectrum of somatic gene mutations in the KLK6-high and KLK6-low groups by analyzing the available TCGA DNA sequencing results. As demonstrated in Table 1 and Figure S2A, (based on Table S1A data), the most frequent cancer-related mutations observed in KLK6-high group were mutations in the *TTN* and *APC* genes (found in 75% of KLK6-high tumors), followed by mutations in *K-RAS* oncogene (68.75%) and *MUC16* (mutated in 56.25%). The mutations in *p53* tumor suppressor gene were detected in half samples with high *KLK6* expression (Table 1).

In contrast, the *APC* mutations were identified in all *KLK6* -low expressing tumors (100%) and 66.67% of tumors in this group had p53 mutations. No mutations in *TTN* or *K-RAS* were detected in KLK6-low group (Table 1, Figure S2B and Table S1B).

We also assessed the status of *KLK6* gene in KLK6-high and KLK6-low samples and did not find any changes in *KLK6* gene copy number in these groups. Overall, twenty-five tumors within the remaining 469 cases of the TCGA cohort had alteration in *KLK6* gene, with 80% of the cases carrying the missense mutations in *KLK6* coding region and 20% of the cases having deletions in the 3′UTR or splice region of *KLK6* (Table S1C). This analysis indicates that overexpression of *KLK6* in the CRC samples from the TCGA cannot be attributed to alterations in the *KLK6* gene copy number or gene’s structural aberrations.

### 3.3. Survival Analysis

**Figure 2:**
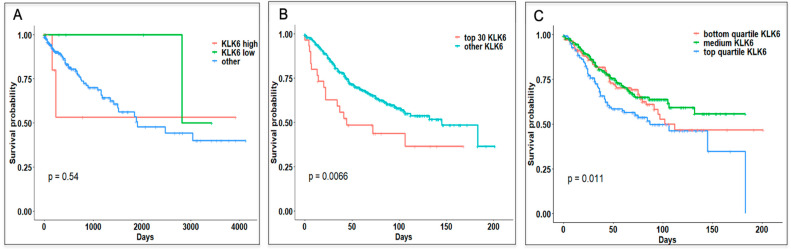
Overall Survival analysis of patients based on KLK6 gene expression from TCGA and GEO datasets. (**A**) Kaplan-Meier Plot of colon adenocarcinoma samples from TCGA. KLK6 High (16 samples), KLK6 low (7 samples), and others were plotted for Overall Survival. (**B**) Kaplan-Meier plot of GEO dataset for KLK6 high samples vs. others. (**C**) Kaplan-Meier plot of GEO dataset: based on KLK6 gene expression, data was plotted for upper quartile, bottom quartile, and intermediate expression for overall survival.

Kaplan-Meier survival curves of patients with high *KLK6* from TCGA demonstrated that they had overall worse survival compared to other patient samples, although the *p* value did not reach significance (Figure 2A). Patients with a significantly low *KLK6* expression (KLK-low group) had no deaths reported. Because of the limited number of samples with the significantly elevated levels of *KLK6* in TCGA, we evaluated the OS rates of the CRC cases from the GEO dataset, GSE39582 of 566 samples. The clinical, histopathological, and molecular characteristics of the samples in this dataset are summarized in Table S2. Analysis of GEO samples with the high *KLK6* expression levels (30 samples with z score >1.96) showed a statistically significant decrease in OS rates for these patients (Figure 2B) compared to all other patients (536 samples) (*p* = 0.0066). Dividing the GEO samples into top and bottom quartiles based on *KLK6* expression also showed that top quartile has worse survival compared to other groups (*p* = 0.01) (Figure 2C).

### 3.4. KLK6 Expression in Left and Right Sided CRC Cases

The right-sided and left-sided colon cancer differ in their clinical and molecular features, and the right-sided colon cases have the worse prognosis [19]. We stratified TCGA CRC cases by the *KLK6* expression in the ascending (right) and descending (left) portions of the colon. The *KLK6* expression was highly variable within the right, left, and transverse colons with no significant difference between tumor locations. The expression of *KLK6* was significantly higher in all tumor areas compared to the normal tissues (*p* = 0.001, normal vs. descending tumors, *p* = 1.6 × 10^−6^, normal vs. ascending tumors) (Figure S3).

### 3.5. Differential Analysis of Samples with Overexpressed KLK6

**Figure 3:**
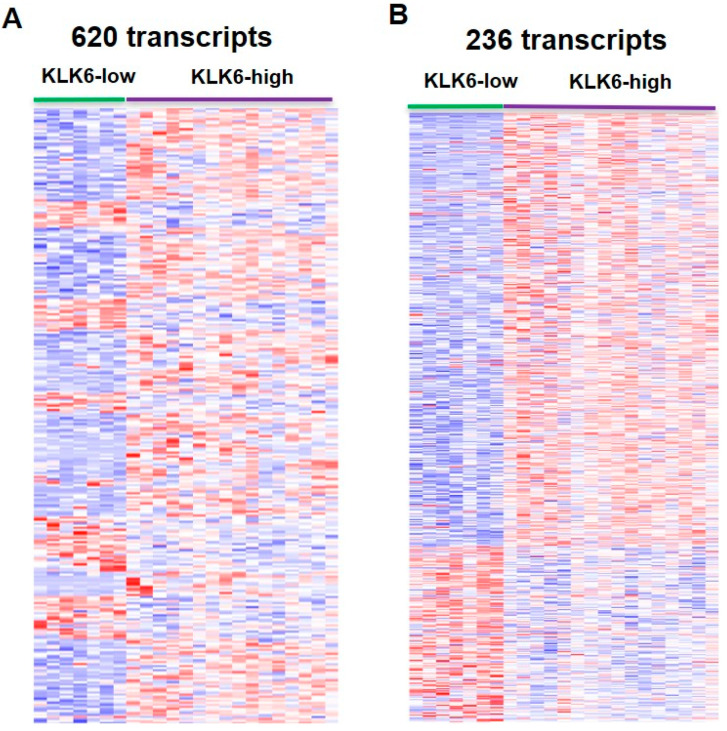

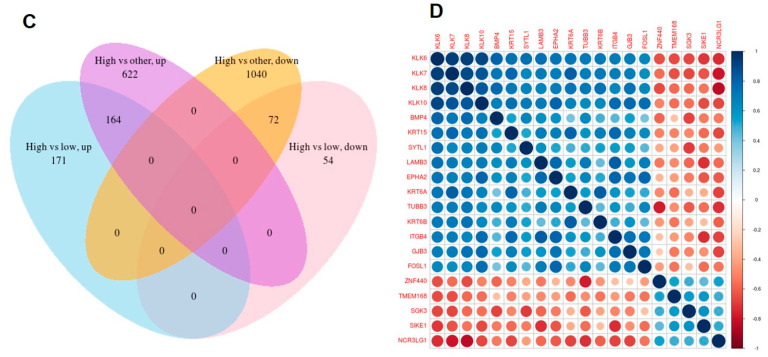
Clustering of the colon adenocarcinoma TCGA samples with differential expression of KLK6. (**A**) Heatmap showing differentially expressed protein-coding genes in KLK6-high vs. KLK6-low group samples. (**B**) Heatmap showing a group of 236 differentially expressed protein-coding genes, which are common in high KLK6 vs. low KLK6 group and high KLK6 vs. all other samples. (**C**) Venn diagram showing number of genes which were found to be up and down when KLK6-high group was compared with all other CRC cases. (**D**) Correlation plot of genes co-expressed with KLK6 in KLK6-high group.

A differential expression analysis was done to assess the differences in gene expression between the KLK6-high and KLK6-low patient samples. DESEQ2 analysis revealed 620 differentially expressed genes (DEGs) between these groups (Table S3A). The DEGs set contained 475 protein coding genes and 145 long non-coding RNAs as well as antisense and uncharacterized transcripts. Cluster analysis of altered gene expression between the KLK6-high and KLK6-low groups showed the KLK6-dependent pattern of gene expression across the groups (Figure 3A). Further differential expression analysis was done between KLK6-high group and the rest of the CRC cases to determine whether any specific genes were altered in KLK6-high group compared to all other samples in the tumor dataset. In the list of all DEGs we identified 236 protein-coding genes which were altered in KLK6-high group (Table S3B). A heatmap of these 236 genes, shown in Figure 3B, demonstrates the pattern of KLK6-dependent gene expression in protein coding genes. Depicted in Figure 3C, the Venn diagram demonstrates no overlapping in gene expression between KLK6-high cases and KLK6-low cases, with 171 upregulated genes and 54 downregulated genes in KLK6 high group compared to KLK6-low samples and other samples. Therefore, this set of genes is uniquely attributed to the high KLK6 expression in CRC samples. Correlation analysis across high and low KLK6 groups identified a set of transcripts that strongly correlate with *KLK6* expression and showed the same expression trend (correlation of 0.6 and *p* value < 0.05 were used as significance of association). The correlation plot of top genes, which co-express positively or negatively with *KLK6* overexpression, is shown in Figure 3D. The highly associated genes include the members of kallikrein family (*KLK7, KLK8, KLK10,* and *KLK11*), keratins (*KRT80, KRT6A, KRT6B, KRT19, KRT15, KRT19*), extracellular matrix proteins, vesicle-mediated protein trafficking, and signaling genes, e.g., *BMP* (Bone Morphologic Protein 4), and *GJB3* (Gap Junction protein beta 3) (Table S3C). 

Differential expression analysis of KLK6-high samples (30 samples) and other samples (536) in GEO dataset (total of 566 samples) identified 691 significantly altered genes. When we compared the set of 236 altered genes in KLK6-high group from TCGA dataset with 691 genes from GEO dataset, we found 121 common genes (Table S3D).

### 3.6. Pathways and Significant Cellular Functions That Associated with KLK6 Overexpression

**Figure 4:**
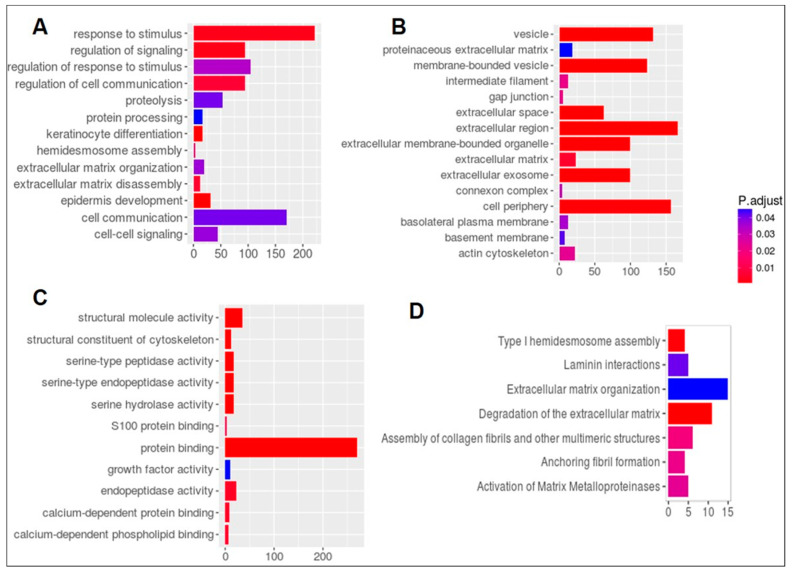
Analysis of Gene Ontology (GO) and signaling pathways associated with KLK6 overexpression in TCGA colon adenocarcinoma samples. Analysis of DEGs for GO shows (**A**) Biological processes. (**B**) Cellular Components and (**C**) Molecular Functional gene sets that are significantly enriched in high expressing KLK6 group. (**D**) Reactome pathways that are enriched in DEGs.

We next applied Gene Ontology (GO) and pathway enrichment analyses to find which GO terms are over-represented and under-represented in TCGA KLK6-high DEGs set. In the KLK6-high group we found the most significant association (*p* value < 0.05) of GO terms for biological process (Figure 4A) with the genes involved in response to stimulus, regulation of signaling, and regulation of cell communication. Figure 4B demonstrates significantly enriched GO cellular component genes in KLK6-high group involving the plasma membrane, both basolateral and basement, gap junctions, and connexon complex, as well as membrane-bound vesicle and extracellular exosome. Protein binding genes and peptidase activity (Figure 4C) were significantly enriched for GO molecular function terms (Table S4A).

Reactome pathways analysis [20] was applied to search for pathways and several gene sets in Type I hemidesmosome assembly, Laminin interactors, extracellular matrix organization, degradation of the extracellular matrix, assembly of collagen fibrils, Anchoring fibril formation, and Activation of Matrix metalloproteinases were found to be differentially enriched in KLK6 high expression samples (Figure 4D, Table S4B).

**Figure 5:**
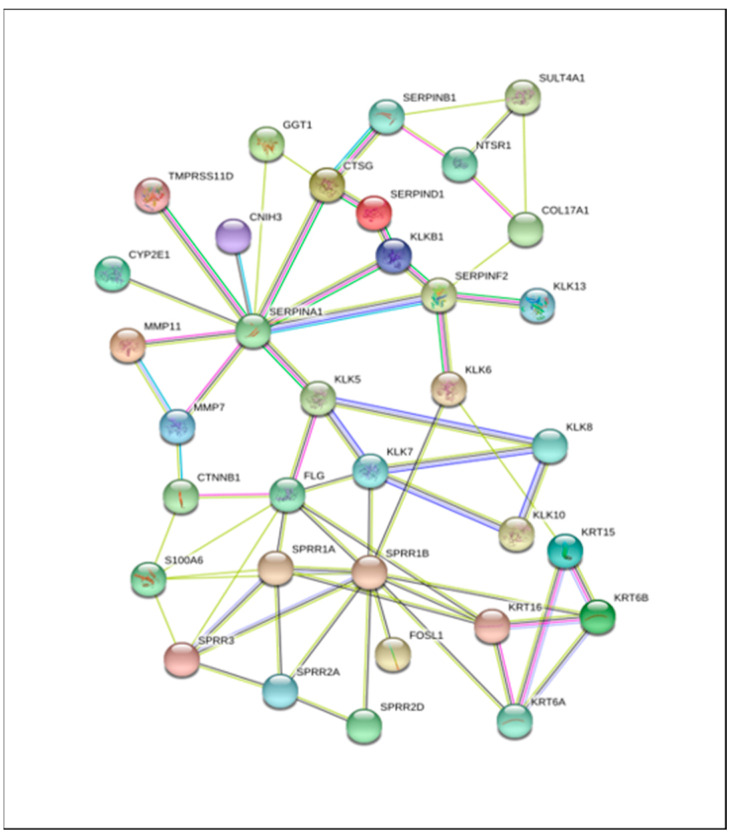
Protein Interaction network of kallikriens and differentially expressed genes found in high KLK6 samples using Stringdb interaction dataset.

Finally, the network of kallikreins interacting genes (*KLK6, 7, 8, 10, 13*) and their first interacting partners was developed using Stringdb (Figure 5 and Table S4C). We report here the top 34 extracellular matrix proteins with protein binding function. The list includes metalloproteases, serine proteases, keratins, and proteins involved in keratin differentiation (Table S4D).

### 3.7. Analysis of Expression and Secretion of KLK6 in the CRC Patient-Derived Organoid Cultures

**Figure 6:**
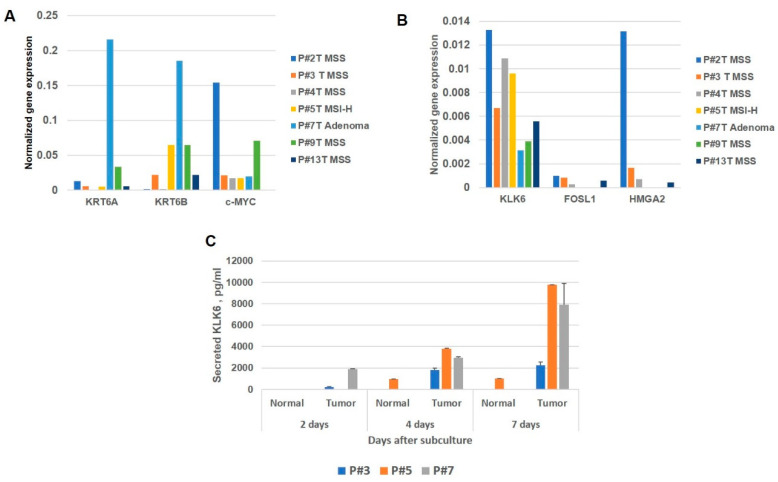
Evaluation of the transcript levels of selected co-expressed genes and KLK6 secretion in the CRC patient-derived organoid cultures. (**A**) KRT6A, KRT6B and c-MYC transcript levels. (**B**) *KLK6, FOSL1*, and *HMGA2* transcript levels. (**C**) Levels of secreted KLK6 in the conditioned media of the tumor organoid cultures by ELISA.

We analyzed RNA levels of *KLK6* and the selected co-expressed genes from the set depicted in a correlation plot (Figure 3D) in the paired normal and tumor organoid cultures. The organoid cultures were established from the surgical material (paired normal tissue and primary tumor) and collected from the consent treatment-naïve CRC patients according to the Institutional Tissue Collection Protocol. Clinicopathological characteristics of the tumors processed for organoid cultures are presented in Table S5. We found that *KLK6* RNA levels vary significantly in the screened cases regardless of the molecular subtype of the analyzed tumors (Figure 6A). The *KRT6A* and *KRT6B* gene transcripts were significantly elevated in the adenoma (one case) compared to malignant tumors (Figure 6B). We also assessed the expression patterns of *c-MYC* oncogene and *HMGA2* transcription factor in the organoid cultures, because of the important role of *c-MYC* oncogene in colon tumorigenesis [21] and the association of *HMGA2* with the poor disease outcome and *KLK6* overexpression in the CRC patients [22,23]. The *c-MYC* transcript levels were significantly higher in P#2T and P#13T organoid cultures (both cases had MSS tumor subtype and stage T3N0). These samples also had the high levels of *KLK6*, *HMGA2*, and *FOSL1* transcripts, which may indicate the aggressive phenotype of these tumors. The *HMGA2* and *FOSL1* transcript levels were detected in 4 out of 7 cases. We also assessed the secreted KLK6 in the conditioned media of organoids derived from the normal and tumor tissues. The analysis of the secreted KLK6 levels was done in three cases, representing the colon cancer samples (P#3 and P#5) and a colon adenoma (P#7). The elevated levels of secreted KLK6 were detected in the tumor cultures compared to normal ones and they increased with the time in culture (Figure 6C). The secreted KLK6 was below the level of detection in the normal cultures of P#3 and P#7. The detected secreted KLK6 in the normal culture of P#5 suggests that the tissue has acquired some invasive properties. Overall, this pilot analysis confirms the variability of KLK6 expression in the CRC and demonstrates the experimental platform for more detailed analysis of key genes in the colon cancer. 

## 4. Discussion

The ongoing challenge in cancer treatment is to identify the most appropriate targeted therapy for managing the disease based on the individual properties of the tumor. This precision medicine approach requires identification of patients’ individual genetic, epigenetic, and other alterations for further stratification into subgroups with matching drug treatment options. Kallikreins have become a subject of intense investigation as prognostic markers and candidates for drug targeting because of their important functions in normal physiology and pathological state, which have been discovered in the past years (reviewed in [24]). Recent analysis of kallikrein gene family across 15 different cancers highlighted KLK6, KLK7, KLK8, and KLK10 as the excellent diagnostic biomarkers candidates for colon adenocarcinoma [25]. Another analysis of TCGA dataset identified *KLK6* gene and *c-MYC* oncogene as the two overexpressed genes, which are significantly associated with the overall survival in patients and, therefore, highly accurate in predicting the disease outcome [26]. 

In our study, KLK6-high CRC cases in TCGA revealed that 25% of patients in this group had an advanced clinical stage (Stage IV and IVA), while no patients in the KLK6-low group were diagnosed with Stage IV of the disease. No difference in the molecular subtypes of the adenocarcinomas were noted between KLK6-high and KLK6-low cases. At the same time, the analysis of the mutations revealed that the KLK6-high cases had the high frequencies of mutations in *TTN, APC, K-RAS,* and *MUC16* genes, while in the KLK6-low group only *APC* mutations (found at 100% frequency) and *TP53* mutation (identified in 66% of cases) were prevalent. We have previously reported the mutant *K-RAS*-dependent expression of *KLK6* gene in the colon cancer cells [11,27], so the current results from TCGA patient data confirmed the validity of the previous findings. 

The mutational landscape in the KLK6-high group suggests the possibility of utilizing the tumor mutational burden (TMB) biomarker for estimating the therapy outcomes in the CRC patients with high *KLK6* expression. The TMB is a novel biomarker, which is utilized for predicting responsiveness to Immune Checkpoint Blockage (ICB) in CRC patients with MSI-H tumors [28,29]. Here we found that KLK6 high tumors carry mutations in two longest genes in the human genome, i.e., *TTN*, a structural protein in striated muscles [30] and *MUC16*, which encodes the cancer antigen CA-125. The correlation between the high TMB and the mutational status of the large size genes has been previously suggested [31]. Recent analysis of a discovery cohort of 34 solid tumors and 7 cohorts from clinical trials treated with programmed cell-death ligand 1 (PD-L1) inhibitor demonstrated that *TTN* mutations in solid tumors can be used to stratify into groups with different clinical responses to ICB monotherapy [31]. The unique presence of *TTN* and *MUC16* mutations along with the high frequency of *K-RAS* and *APC* mutations in the KLK6-high cases from TCGA dataset warrants further validation of this set of mutations for selecting the therapy type for the patients with *KLK6* overexpression in prospective clinical trials. The phosphoinositidine 3-kinase (*PIK3CA*) is another commonly mutated gene in CRC and it was found in ~43% of the KLK6-high tumors. Our correlation analysis identified the members of kallikrein family, *KLK7, KLK8, KLK10*, as the most highly co-expressed with *KLK6*. Of interest, *KLK6* and *KLK10* overexpression and co-expression has been reported in pancreatic adenocarcinomas as an unfavorable factor associated with a poor OS [32]. 

The set of upregulated keratins, i.e., 6A, 6B, 15, 16, 19, 80, in the KLK6-high CRC tumors was different from the one, identified in KLK6-overexpressing serous ovarian cancer [33]. This finding suggests that KLK6 regulates expression of keratins in cancer in a tissue-specific manner. The list of altered genes also included the extracellular matrix proteins *ING4B* (integrin 4B), *LAMB3* (laminin subunit beta3), vesicle-mediated transport protein coding genes *SYL1* (Synaptotagmin Like 1), and *GJB3*, as well as signaling genes, *BMP4* in TGF-β signaling pathway and transcription factor *FOSL1* (FOS-like transcription factor subunit). Analysis of the functional roles of the most altered genes showed that they are important structural components of the cell cytoskeleton, gap junction, and connexon complexes, the basolateral membrane and actin cytoskeleton, as well as extracellular exosomes, which indicates that KLK6 overexpression contributes to aggressive growth and invasion in tumor cells.

Protein interaction network for KLK6-overexpressing cases allowed us to identify proteins which may interact with KLK6, either through formation of protein complexes or via modifications of other proteins. Particularly, the presence of the kallikrein family members, KLK5, KLK7, KLK8, KLK10, KLK13, and the metalloproteinase family members MMP7 and MMP11 in this network suggests that active KLK6 enzyme may induce activation and cross activation of other proteases. The constructed network predicts the direct interactions between KLK7, KLK8, and KLK10, but not KLK6. This observation highlights complex relations among kallikreins in the CRC. We found keratins 6A, 6B, 15, and 16, as well as Small Proline Rich Repeat (*SPRR*) genes (*SPRR1A, SPRR1B, SPRR2A, SPRR2D,* and *SPRR3*), which are involved in the keratinization pathway, as interacting partners of KLK6. This suggests the mechanism of KLK6-mediated upregulation of a subgroup of keratin genes in KLK6-high group. Keratins are established epithelial cell markers and can regulate multiple signaling pathways in cancer. The keratins altered in KLK6-high colorectal tumors may be further evaluated as biomarkers in the CRC progression. SPRRs proteins are the keratinocytes differentiation markers, involved in formation of the cornified envelope in skin cells, but their function in cancer is not well defined. The SPRR3 has been reported as tumor suppressor in esophageal squamous carcinoma cells [34]. The S100A6 protein, which was found to be an interacting partner of SPRR3 and SPRR1A in KLK6 high group, has been associated with cells motility and tumor metastasis in cervical cancer cells and lung carcinoma [35,36]. SERPIN F2, a serine protease inhibitor, which was identified in connection with KLK6 overexpression, is a known inhibitor of plasmin, trypsin, and chemotrypsin.

Although in this study we focused on protein-coding genes, we noted top two highly up regulated non-coding transcripts in KLK6 high group samples, i.e., *CTB-147C22.8* and *CTB147C22*, which were reported to be co-expressed with kallikrein genes *KLK5, KLK6*, and *KLK7* in HPV16^+^ head and neck tumors [37]. Using a set of 7 patient derived tumor organoid cultures, we confirmed the variability in expression and secretion of KLK6, and the necessity of an individualized approach in designing the CRC chemotherapy. 

The findings presented here suggest that KLK6-high tumors may be sensitive to the specific inhibitors of kallikreins. The reversible inhibitors of the members of kallikrein family have been previously reported [38,39,40]. Applicable to our targets, the promising inhibitors of KLK6 [41,42] and KLK7 [43] have been recently characterized. The pre-clinical testing of selected kallikrein inhibitors should be done to validate their use as investigational drugs in clinical trials, especially because of their demonstrated high complexity of interactions between kallikreins. 

The analysis of *KLK6*-overexpressing cases from TCGA was verified using a GEO CRC sample set and the overall findings were in agreement with the results of TCGA analysis. There were higher numbers of *KLK6*-overexpression cases in this set (30 samples vs. 16 in TCGA) and the GEO patients had more advanced stages of the disease (no patients with Stage 1 and the higher percent of patients had Stage II and Stage III of the disease). Perhaps because of these differences, the analysis of OS rates in the GEO dataset showed that patient’s survival rates significantly correlated with KLK6 overexpression in tumors, while in TCGA samples the Kaplan-Meier curves demonstrated a similar trend.

## 5. Conclusions

In this study we discovered a distinct set of genes associated with high *KLK6* expression in CRC samples. The identified families of genes, encoding keratins, extracellular matrix proteins, protein trafficking and TGF-β, FOS, and Ser/Thr protein kinase proteins contribute to tumor progression, cell invasion, and metastasis in *KLK6*-overexpressed CRCs. Our analysis suggests that identifying colon cancer patients with KLK6 overexpression and targeting it using specific inhibitors can be beneficial for the suppression of metastasis. In future studies we hope to validate these genes and pathways with cellular assays in organoids from relevant patient samples and to evaluate the targeted treatment with investigational drugs. 

## Figures and Tables

**Table 1:** Clinical and molecular analysis of the KLK6-high and KLK6-low groups in TCGA colon adenocarcinoma dataset.

Clinical, Pathological and Molecular Characteristics		Groups	
		KLK6-High Group (*n* = 16), % (Number of Cases/Group)	KLK6-Low Group (*n* = 7), % (Number of Cases/Group)
Gender	female	44 (7/16)	71 (5/7)
	male	56 (9/16)	29 (2/7)
Tumor stage	Stage I	19 (3/16)	28.6 (2/7)
	Stage II A	37 (6/16)	28.6 (2/7)
	Stage II B	0 *	14. 29 (1/7)
	Stage III	6.25 (1/16)	0
	Stage III B	6.25 (1/16)	14.29 (1/7)
	Stage III C	6.25 (1/16)	14.29 (1/7)
	Stage IV	19 (3/16)	0
	Stage IV A	6.25 (1/16)	0
Metastasis ≥M1		25 (4/16)	0
Lymph node positive		44 (7/16)	28.6 (2/5)
Molecular subtype **	MSS	43.75 (7/16)	42.85 (3/7)
	MSI-L	31.25 (5/16)	14.2 (1/7)
	MSI-H	25 (4/16)	42.85 (3/7)
Mutations ***	*APC*	75 (12/16)	100 (6/6)
	Titin (*TTN*)	75 (12/16)	0
	*K-RAS*	68.75(11/16)	0
	*MUC16*	56.25 (9/16)	0
	*TP53*	50 (8/16)	66.67 (4/6)

* no cases found within a group. ** MSS-microsatellite stable; MSI-microsatellite instable; MSH-H- MSI-high; MSI-L-MSI-low. *** shown only the top 5 mutations per group.

## Data Availability

All data generated and analyzed during this study are included in the published article and the supplementary information files.

## Supplementary Materials

The following are available online at https://www.mdpi.com/article/10.3390/genes12050749/s1, Figure S1. KLK6 expression in normal colon and tumors from TCGA stratified according to Consensus Molecular subtypes (CMS). CMS1-4 are four subtypes defined as in [18]. NoLBL are TCGA tumors with no labels or could not be subtype classified. Figure S2. Mutation frequencies in the colon adenocarcinoma TCGA samples with the differential KLK6 transcript level. (A) KLK6-high group, (B) KLK6-low group. Figure S3. Analysis of KLK6 expression in the right side, left side and transverse CRC cases from TCGA. Box plot shows distribution of KLK6 expression across site of colon adenocarcinoma tumor and normal samples. Table S1. Supplemental data for molecular characterization of TCGA samples. Data are from Genomics Data Commons (GDC). (A) Top 100 frequently mutated genes in Colon adenocarcinoma TCGA KLK6 high samples. (B) Top 100 mutated genes in Colon adenocarcinoma TCGA low KLK6 samples. (C) Analysis of alterations in KLK6 gene in TCGA cohort. Table S2. Patient Characteristics of high KLK6 samples from GEO dataset GSE39582. Table S3. Differential expression analysis of genes in the KLK6-high and KLK6-low patient samples. (A) Differential Expressed Genes between high KLK6 expressed Group and low KLK6 expressed groups in TCGA Colon Adenocarcinoma samples; (B) 236 Differential Expressed Genes between high KLK6 expressed Group and low KLK6 expressed groups and high KLK6 expressed groups and rest all the samples in TCGA colon tumor; (C) Genes correlated with KLK6 in Colon adenocarcinoma TCGA samples. (D) Genes found in common between TCGA DEG list of 236 genes and GEO dataset GSE39582 of KLK6 high samples compared with rest of the samples. Table S4. Supplemental data for pathways associated with KLK6 overexpression in colorectal cancer. (A) Gene Ontology Terms enriched in KLK6 high samples in TCGA for Figure 6C. (B) Reactome Pathways enriched in KLK6 high samples in TCGA for Figure 6D. (C) Protein Interaction of Kallikreins and differential expression genes in KLK6 High Samples in TCGA from Stringdb for Figure 5. (D) Annotation-Protein Interaction partners with Kallikreins in Figure 5. Table S5. Clinicopathological characteristics of surgical cases established as organoid cultures.

## References

[1] Cavallo F., De Giovanni C., Nanni P., Forni G., Lollini P.L. (2011). 2011: The immune hallmarks of cancer. Cancer Immunol Immunother..

[2] Sotiropoulou G., Pampalakis G., Diamandis E.P. (2009). Functional roles of human kallikrein-related peptidases. J. Biol. Chem..

[3] Devetzi M., Trangas T., Scorilas A., Xynopoulos D., Talieri M. (2013). Parallel overexpression and clinical significance of kallikrein-related peptidases 7 and 14 (KLK7KLK14) in colon cancer. Thromb Haemost..

[4] Vakrakou A., Devetzi M., Papachristopoulou G., Malachias A., Scorilas A., Xynopoulos D., Talieri M. (2014). Kallikrein-related peptidase 6 (KLK6) expression in the progression of colon adenoma to carcinoma. Biol. Chem..

[5] Alexopoulou D.K., Papadopoulos I.N., Scorilas A. (2013). Clinical significance of kallikrein-related peptidase (KLK10) mRNA expression in colorectal cancer. Clin. Biochem..

[6] Alexopoulou D.K., Kontos C.K., Christodoulou S., Papadopoulos I.N., Scorilas A. (2014). KLK11 mRNA expression predicts poor disease-free and overall survival in colorectal adenocarcinoma patients. Biomark Med..

[7] Borgono C.A., Diamandis E.P. (2004). The emerging roles of human tissue kallikreins in cancer. Nat. Rev. Cancer.

[8] Ogawa K., Utsunomiya T., Mimori K., Tanaka F., Inoue H., Nagahara H., Murayama S., Mori M. (2005). Clinical significance of human kallikrein gene 6 messenger RNA expression in colorectal cancer. Clin. Cancer Res..

[9] Ohlsson L., Lindmark G., Israelsson A., Palmqvist R., Oberg A., Hammarstrom M.L., Hammarstrom S. (2012). Lymph node tissue kallikrein-related peptidase 6 mRNA: A progression marker for colorectal cancer. Br. J. Cancer.

[10] Fearon E.R. (2011). Molecular genetics of colorectal cancer. Annu. Rev. Pathol..

[11] Henkhaus R.S., Gerner E.W., Ignatenko N.A. (2008). Kallikrein 6 is a mediator of K-RAS-dependent migration of colon carcinoma cells. Biol. Chem..

[12] Sells E., Pandey R., Chen H., Skovan B.A., Cui H., Ignatenko N.A. (2017). Specific microRNA-mRNA Regulatory Network of Colon Cancer Invasion Mediated by Tissue Kallikrein-Related Peptidase 6. Neoplasia.

[13] Anders S., Huber W. (2010). Differential expression analysis for sequence count data. Genome Biol..

[14] Love M.I., Huber W., Anders S. (2014). Moderated estimation of fold change and dispersion for RNA-seq data with DESeq2. Genome Biol..

[15] Szklarczyk D., Gable A.L., Lyon D., Junge A., Wyder S., Huerta-Cepas J., Simonovic M., Doncheva N.T., Morris J.H., Bork P. (2019). STRING v11: Protein-protein association networks with increased coverage, supporting functional discovery in genome-wide experimental datasets. Nucleic Acids Res..

[16] Sato T., Stange D.E., Ferrante M., Vries R.G., Van Es J.H., Van den Brink S., Van Houdt W.J., Pronk A., Van Gorp J., Siersema P.D. (2011). Long-term expansion of epithelial organoids from human colon, adenoma, adenocarcinoma, and Barrett’s epithelium. Gastroenterology.

[17] Cancer Genome Atlas Network (2012). Comprehensive molecular characterization of human colon and rectal cancer. Nature.

[18] Guinney J., Dienstmann R., Wang X., de Reynies A., Schlicker A., Soneson C., Marisa L., Roepman P., Nyamundanda G., Angelino P. (2015). The consensus molecular subtypes of colorectal cancer. Nat. Med..

[19] Lee M.S., Menter D.G., Kopetz S. (2017). Right Versus Left Colon Cancer Biology: Integrating the Consensus Molecular Subtypes. J. Natl. Compr. Cancer Netw..

[20] Fabregat A., Sidiropoulos K., Garapati P., Gillespie M., Hausmann K., Haw R., Jassal B., Jupe S., Korninger F., McKay S. (2016). The Reactome pathway Knowledgebase. Nucleic Acids Res..

[21] Ignatenko N.A., Holubec H., Besselsen D.G., Blohm-Mangone K.A., Padilla-Torres J.L., Nagle R.B., de Alboranc I.M., Guillen R.J., Gerner E.W. (2006). Role of c-Myc in intestinal tumorigenesis of the ApcMin/+ mouse. Technol. Cancer Res. Treat..

[22] Morishita A., Zaidi M.R., Mitoro A., Sankarasharma D., Szabolcs M., Okada Y., D’Armiento J., Chada K. (2013). HMGA2 is a driver of tumor metastasis. Cancer Res..

[23] Chen H., Sells E., Pandey R., Abril E.R., Hsu C.H., Krouse R.S., Nagle R.B., Pampalakis G., Sotiropoulou G., Ignatenko N.A. (2019). Kallikrein 6 protease advances colon tumorigenesis via induction of the high mobility group A2 protein. Oncotarget.

[24] Prassas I., Eissa A., Poda G., Diamandis E.P. (2015). Unleashing the therapeutic potential of human kallikrein-related serine proteases. Nat. Rev. Drug Discov..

[25] Tailor P.D., Kodeboyina S.K., Bai S., Patel N., Sharma S., Ratnani A., Copland J.A., She J.X., Sharma A. (2018). Diagnostic and prognostic biomarker potential of kallikrein family genes in different cancer types. Oncotarget.

[26] Dong S., Ding Z., Zhang H., Chen Q. (2019). Identification of Prognostic Biomarkers and Drugs Targeting Them in Colon Adenocarcinoma: A Bioinformatic Analysis. Integr. Cancer Ther..

[27] Ignatenko N.A., Zhang H., Watts G.S., Skovan B.A., Stringer D.E., Gerner E.W. (2004). The chemopreventive agent alpha-difluoromethylornithine blocks Ki-ras-dependent tumor formation and specific gene expression in Caco-2 cells. Mol. Carcinog..

[28] Yarchoan M., Hopkins A., Jaffee E.M. (2017). Tumor Mutational Burden and Response Rate to PD-1 Inhibition. N. Engl. J. Med..

[29] Samstein R.M., Lee C.H., Shoushtari A.N., Hellmann M.D., Shen R., Janjigian Y.Y., Barron D.A., Zehir A., Jordan E.J., Omuro A. (2019). Tumor mutational load predicts survival after immunotherapy across multiple cancer types. Nat. Genet..

[30] Ciferri A., Crumbliss A.L. (2018). The Assembling and Contraction Mechanisms of Striated Muscles. Front. Chem..

[31] Jia Q., Wang J., He N., He J., Zhu B. (2019). Titin mutation associated with responsiveness to checkpoint blockades in solid tumors. JCI Insight.

[32] Ruckert F., Hennig M., Petraki C.D., Wehrum D., Distler M., Denz A., Schroder M., Dawelbait G., Kalthoff H., Saeger H.D. (2008). Co-expression of KLK6 and KLK10 as prognostic factors for survival in pancreatic ductal adenocarcinoma. Br. J. Cancer.

[33] Wang P., Magdolen V., Seidl C., Dorn J., Drecoll E., Kotzsch M., Yang F., Schmitt M., Schilling O., Rockstroh A. (2018). Kallikrein-related peptidases 4, 5, 6 and 7 regulate tumour-associated factors in serous ovarian cancer. Br. J. Cancer.

[34] Zhang Y., Feng Y.B., Shen X.M., Chen B.S., Du X.L., Luo M.L., Cai Y., Han Y.L., Xu X., Zhan Q.M. (2008). Exogenous expression of Esophagin/SPRR3 attenuates the tumorigenicity of esophageal squamous cell carcinoma cells via promoting apoptosis. Int. J. Cancer.

[35] Li A., Gu Y., Li X., Sun H., Zha H., Xie J., Zhao J., Huang M., Chen L., Peng Q. (2018). S100A6 promotes the proliferation and migration of cervical cancer cells via the PI3K/Akt signaling pathway. Oncol. Lett..

[36] Li P., Lv X., Zhang Z., Xie S. (2019). S100A6/miR193a regulates the proliferation, invasion, migration and angiogenesis of lung cancer cells through the P53 acetylation. Am. J. Transl. Res..

[37] Salyakina D., Tsinoremas N.F. (2016). Non-coding RNAs profiling in head and neck cancers. NPJ Genom. Med..

[38] Goettig P., Magdolen V., Brandstetter H. (2010). Natural and synthetic inhibitors of kallikrein-related peptidases (KLKs). Biochimie.

[39] Liang G., Chen X., Aldous S., Pu S.F., Mehdi S., Powers E., Giovanni A., Kongsamut S., Xia T., Zhang Y. (2012). Virtual Screening and X-ray Crystallography for Human Kallikrein 6 Inhibitors with an Amidinothiophene P1 Group. ACS Med. Chem. Lett..

[40] Sotiropoulou G., Pampalakis G. (2012). Targeting the kallikrein-related peptidases for drug development. Trends Pharm. Sci..

[41] Sananes A., Cohen I., Shahar A., Hockla A., De Vita E., Miller A.K., Radisky E.S., Papo N. (2018). A potent, proteolysis-resistant inhibitor of kallikrein-related peptidase 6 (KLK6) for cancer therapy, developed by combinatorial engineering. J. Biol. Chem..

[42] De Vita E., Schuler P., Lovell S., Lohbeck J., Kullmann S., Rabinovich E., Sananes A., Hessling B., Hamon V., Papo N. (2018). Depsipeptides Featuring a Neutral P1 Are Potent Inhibitors of Kallikrein-Related Peptidase 6 with On-Target Cellular Activity. J. Med. Chem..

[43] De Veer S.J., Furio L., Swedberg J.E., Munro C.A., Brattsand M., Clements J.A., Hovnanian A., Harris J.M. (2017). Selective Substrates and Inhibitors for Kallikrein-Related Peptidase 7 (KLK7) Shed Light on KLK Proteolytic Activity in the Stratum Corneum. J. Investig. Derm..

