# Supplementary material for: Molecular Pathways Associated with Kallikrein 6 Overexpression in Colorectal Cancer
Source: Genes (Basel). 2021 May 16;12(5):749. doi: 10.3390/genes12050749 (PMC8157155; doi:10.3390/genes12050749)

S1 Figure. KLK6 expression in normal colon and tumors from TCGA stratified according to Consensus Molecular subtypes (CMS). CMS1-4 are four subtypes defined as in [18]. NoLBL are TCGA tumors with no labels or could not be subtype classified.

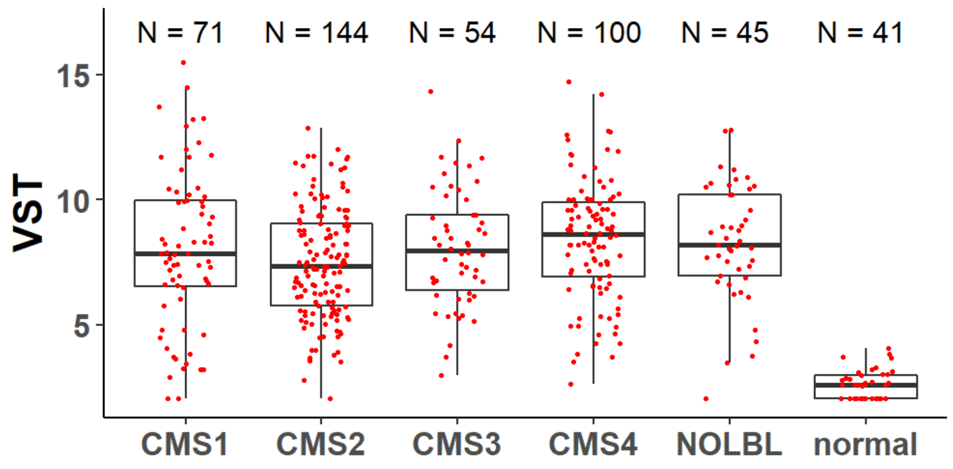

Supplement: Supplementary file 1 [file genes-12-00749-s001.zip › S1 Figure.pdf]
