# Supplementary material for: Molecular Pathways Associated with Kallikrein 6 Overexpression in Colorectal Cancer
Source: Genes (Basel). 2021 May 16;12(5):749. doi: 10.3390/genes12050749 (PMC8157155; doi:10.3390/genes12050749)

S2 Figure. Mutation frequencies in the colon adenocarcinoma TCGA samples with the differential KLK6 transcript level. (A) KLK6-high group, (B) KLK6-low group.

A

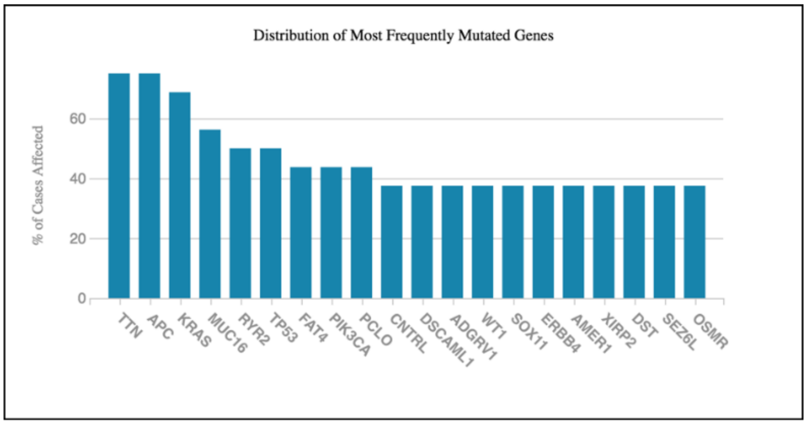

B

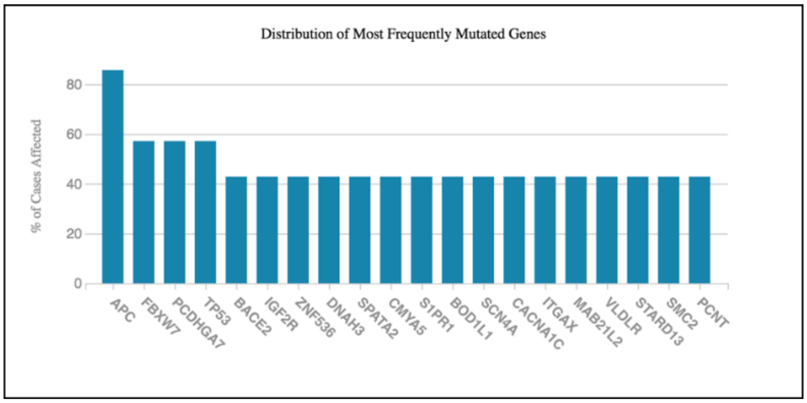

Supplement: Supplementary file 1 [file genes-12-00749-s001.zip › S2 Figure.pdf]
