# Supplementary material for: Molecular Pathways Associated with Kallikrein 6 Overexpression in Colorectal Cancer
Source: Genes (Basel). 2021 May 16;12(5):749. doi: 10.3390/genes12050749 (PMC8157155; doi:10.3390/genes12050749)
Supplement: Supplementary file 1 [file genes-12-00749-s001.zip › S2 Table-rev.pdf]

S2 Table. Patient Characteristics of high KLK6 samples from GEO dataset GSE39582.

Clinical, pathological and molecular characteristics		KLK6-high group (n=30), % cases per group
Gender	female	40
	male	60
Tumor stage	Stage I	0*
	Stage II	53.3
	Stage II A/ B	NA
	Stage III	29
	Stage III B	0
	Stage III C	0
	Stage IV	16.7
	Stage IV A	NA
Metastasis ≥M1		16.6
Lymph node positive		0
Molecular subtype**	MSS	73.3
	MSI-L/H	0
	MSI	16.67
Mutations***	APC	0
	Titin (TTN)	0
	K-RAS	53.3
	MUC16	NA
	P53	52.3

* no cases found within a group
**MSS-microsatellite stable; MSI-microsatellite instable; MSH-H- MSI-high; MSI-L - MSI-low.
*** shown only the top 5 mutations per group
