# Supplementary material for: Molecular Pathways Associated with Kallikrein 6 Overexpression in Colorectal Cancer
Source: Genes (Basel). 2021 May 16;12(5):749. doi: 10.3390/genes12050749 (PMC8157155; doi:10.3390/genes12050749)

S3 Figure. Analysis of KLK6 expression in the right side, left side and transverse CRC cases from TCGA. Box plot shows distribution of KLK6 expression across site of colon adenocarcinoma tumor and normal samples.

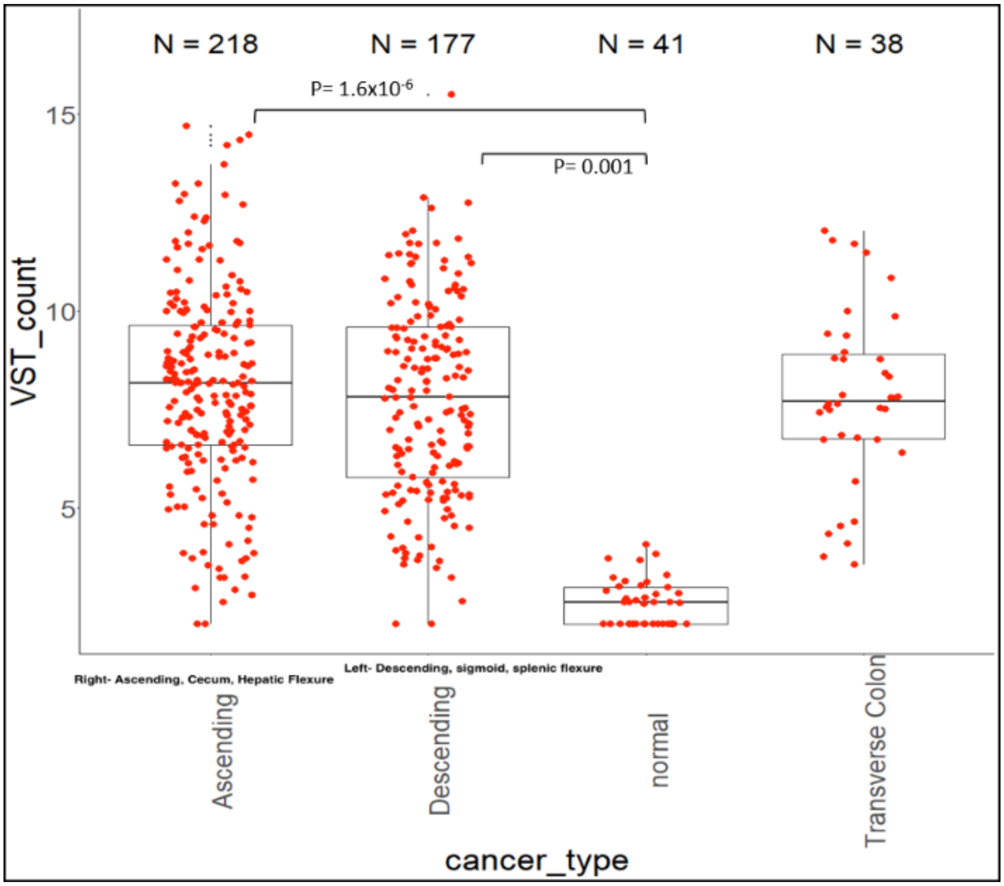

Supplement: Supplementary file 1 [file genes-12-00749-s001.zip › S3 Figure.pdf]
