# Supplementary material for: Molecular Pathways Associated with Kallikrein 6 Overexpression in Colorectal Cancer
Source: Genes (Basel). 2021 May 16;12(5):749. doi: 10.3390/genes12050749 (PMC8157155; doi:10.3390/genes12050749)
Supplement: Supplementary file 1 [file genes-12-00749-s001.zip › S5 Table.pdf]

S5 Table. Clinicopathological characteristics of surgical cases established as organoid cultures

ID	Sex	Molecular subtype	Clinical info
P2	F	MSS	Sigmoid cancer, G2-G3 moderate to poor differentiated, pT3, pN0
P3	F	MSS	Colon and rectal adenocarcinoma, G2 moderately differentiated, pT3, pN0
P4	M	MSS	Rectal adenocarcinoma, G2 moderately differentiated, ypT3N1
P5	M	MSI-H	Transverse colon adenocarcinoma, G2 moderately differentiated, mpT3 pT2 pNo
P7	M	N/A	Ascending colon polyp, Villous adenoma, not malignant
P9	M	MSS	Left colon adenocarcinoma, G2 moderately differentiated, pT2N0
P11	F	MSI-H	Right colon adenocarcinoma, tumor, G1 well differentiated, mpT3 pN0
P12	F	MSS	Colon and rectal adenocarcinoma, G2 moderately differentiated, pT3 pN1b
P13	F	MSS	Right colon adenocarcinoma, G2 moderately differentiated, ypT3N0
