# Supplementary material for: Molecular Pathways Associated with Kallikrein 6 Overexpression in Colorectal Cancer
Source: Genes (Basel). 2021 May 16;12(5):749. doi: 10.3390/genes12050749 (PMC8157155; doi:10.3390/genes12050749)
Supplement: Supplementary file 1 [file genes-12-00749-s001.zip › Supporting information.pdf]

Supporting information

S1 Figure. KLK6 expression in normal colon and tumors from TCGA stratified according to Consensus Molecular subtypes (CMS). CMS1-4 are four subtypes defined as in [18]. NoLBL are TCGA tumors with no labels or could not be subtype classified.

S2 Figure. Mutation frequencies in the colon adenocarcinoma TCGA samples with the differential KLK6 transcript level. (A) KLK6-high group, (B) KLK6-low group.

S3 Figure. Analysis of KLK6 expression in the right side, left side and transverse CRC cases from TCGA. Box plot shows distribution of KLK6 expression across site of colon adenocarcinoma tumor and normal samples.

S1 Table. Supplemental data for molecular characterization of TCGA samples. Data are from Genomics Data Commons (GDC). A) Top 100 frequently mutated genes in Colon adenocarcinoma TCGA KLK6 high samples. **B)** Top 100 mutated genes in Colon adenocarcinoma TCGA low KLK6 samples. **C)** Analysis of alterations in KLK6 gene in TCGA cohort.

S2 Table. Patient Characteristics of high KLK6 samples from GEO dataset GSE39582.

S3 Table. Differential expression analysis of genes in the KLK6-high and KLK6-low patient samples. A) Differential Expressed Genes between high KLK6 expressed Group and low KLK6 expressed groups in TCGA Colon Adenocarcinoma samples; **B)** 236 Differential Expressed Genes between high KLK6 expressed Group and low KLK6 expressed groups and high KLK6 expressed groups and rest all the samples in TCGA colon tumor; **C)** Genes correlated with KLK6 in Colon adenocarcinoma TCGA samples. **D)** Genes found in common between TCGA DEG list of 236 genes and GEO dataset GSE39582 of KLK6 high samples compared with rest of the samples.

S4 Table. Supplemental data for pathways associated with KLK6 overexpression in colorectal cancer. A) Gene Ontology Terms enriched in KLK6 high samples in TCGA for **Fig. 6C. B)** Reactome Pathways enriched in KLK6 high samples in TCGA for **Fig. 6D. C)** Protein Interaction of Kallikreins and differential expression genes in KLK6 High Samples in TCGA from Stringdb for **Fig.7**. **D)** Annotation-Protein Interaction partners with Kallikreins in **Fig. 7**.

S5 Table. Clinicopathological characteristics of surgical cases established as organoid cultures.
